# Traditional therapists in Ebola virus disease outbreak response: Lessons learned from the fight against the Ebola virus disease epidemic in North Kivu and Ituri, Democratic Republic of the Congo

**DOI:** 10.29245/2578-3009/2023/S3.1108

**Published:** 2023-05-12

**Authors:** Nkechi G. Onyeneho, Ngozi Idemili Aronu, Ijeoma Igwe, Joseph Okeibunor, Tieman Diarra, Julienne Ngoudougou Anoko, Mamoudou Harouna Djingarey, Zabulon Yoti

**Affiliations:** 1 University of Nigeria, Nsukka; 2 World Health Organization; 3 Independent Consultant, Mali; 4 Independent Public Health Expert, Niger

**Keywords:** Traditional therapists, Response to the epidemic, Ebola virus disease, Ebola treatment center, Democratic Republic of the Congo

## Abstract

Traditional healers co-exist with orthodox medicine, especially in cases with perceived supernatural causes and during outbreaks of infectious diseases like the Ebola virus disease (EVD) in the North Kivu and Ituri provinces in the Democratic Republic of the Congo (DRC). In this study, we examined the role and potential of involving traditional healers in the national response to the Ebola virus disease outbreak in the DRC. Seventeen community leaders and 20 traditional healers were interviewed. The traditional healers managed symptoms with herbs and were not inclined to refer cases to orthodox healthcare facilities because of their confidence in their ability to handle cases with supernatural causes. The community leaders attested to the acceptance of the traditional healers in the communities, which they attributed to the efficacy of traditional healing, its uncomplicated treatment process, cause of the prolonged cough, as well as cost and the need for secrecy. Traditional healers can be educated to promptly refer cases to Ebola treatment centers for timely diagnosis and appropriate treatment.

## Introduction

Traditional therapists work in most communities in the provinces of North Kivu and Ituri in the Democratic Republic of the Congo (DRC), either individually or in associations. For example, the town of Beni has more than 200 centers that are run by traditional therapists who work within the framework of the Yra Cultural Association. This association is found in two other health zones in the territories of Beni, Mutwenga, and Oicha. The involvement of traditional healers has been varied in these communities and in other communities as reported in the literature^1^. While some have joined the response to infectious disease outbreaks, others have fled their communities owing to fear of stigmatization^1–3^. Traditional healers have been involved in the response to the Ebola virus disease (EVD) epidemic but in different ways depending on the field coordinators. Traditional medicine plays an important role in daily life in the communities in North Kivu and Ituri. Although there were no traditional therapists in some villages involved in this study, all communities use them.

Some traditional healthcare providers have referred patients to an Ebola treatment center (ETC). Traditional therapists are resources that people within the communities rely on in cases of illness^4^. They are often the first to be consulted by the public. Traditional therapists mostly learn their trade in their family, from parents or grandparents. Some are trained in traditional therapists’ associations and others learn their trade after undergoing higher education.

Among the diseases that traditional therapists claim to treat are *Karo, Karufo* (poison), hemorrhoids, sexual dysfunction, sexual impotence, typhoid, liver disease, risk of miscarriage, gynecological issues, witchcraft attacks, childhood diseases, diabetes, paralysis, fractures, panic attacks, sterility, and appendicitis. Some diseases are the exclusive domain of traditional medicine. The involvement of traditional therapists in response to the Ebola epidemic has taken many forms. In some cases, their involvement was facilitated by support from the response teams who initiated and organized it. The response teams trained traditional healers in how to prevent EVD and what to do in the event of a suspected case, that is, referral to an ETC. Traditional healers then adopted behaviors to protect themselves against the disease. In addition to wearing gloves, they installed handwashing amenities in their workplaces.

In other cases, the involvement of traditional therapists was facilitated on an informal basis, based on their personal initiative without contact with the response staff^4^. In these cases, the traditional healers learned about the disease and the means of prevention; they even used the internet for their personal research. They stayed in touch with the referred people, even after they recovered. Apart from referring their patients to a treatment center, some of them conducted activities among the population to raise awareness about the disease and the means of prevention. A traditional therapist in Beni even produced a documentary on EVD, with key messages on prevention and the importance of prompt referral to ETCs.

However, in some cases the response authorities considered the involvement of traditional therapists to be a danger; therefore, they took measures against them, including the closure of their workplaces^4^. Faced with this situation, some traditional therapists left the communities in which they were working, and others transformed their homes into workplaces to hide from the response authorities. They continued to treat patients with suspected EVD; in the course of their work, they were not in contact with the response staff and were not obliged to observe prevention measures. They were not contributing to the response and were not providing opportunities for patients or their relatives to go to an ETC for possible treatment. This situation was not conducive to the response and contributed to concealing potential patients.

However, a striking fact was the behavior of some traditional therapists. They took advantage of the EVD outbreak to engage in self-promotion. They claimed to have developed a remedy for EVD, which they called Eboline. In Beni, response authorities have particularly focused on measures to prevent the spread of opportunistic information by certain traditional therapists.

Against this background, in this study we first focus on the training of traditional therapists and the diseases they treat. We then examine the different forms in which traditional therapists are involved in the EVD response. Finally, proposals by traditional therapists for improving the response are discussed.

## Study Design and Methods

### Study design and sites

This study was designed to explore and document experiences and lessons around the response to the tenth EVD outbreak in the North Kivu and Ituri provinces of the DRC. Qualitative data collection techniques were adopted. The methodology for data gathering included in-depth interviews (IDIs) and focus group discussions. This type of study requires a strong focus on individual rather than state actors^5^.

Ituri is one of the 26 provinces in the DRC; its capital city is Bunia. The Ituri rainforest is in this area. It is located northeast of the Ituri River, on the western side of Lake Albert. Ituri is a high plateau region (2,000–5,000 meters) that has a large tropical forest as well as a savannah landscape. The district has rare fauna, including the okapi, the national animal of the DRC. As for flora, an important species is Mangongo, whose leaves are used by the Mbuti to build their homes. The population is composed primarily of the Alur, Hema, Lendu, Ngiti, Bira, and Ndo-Okebo tribes, with differing figures on which group constitutes the largest percentage. The Mbuti, a pygmy ethnic group, reside primarily in the Ituri rainforest near the Okapi Wildlife Reserve, although some Mbuti have been forced into urban areas by deforestation, over-hunting, and violence. The Kilo-Moto gold mines are partly located in Ituri. At the beginning of the twenty-first century, Heritage Oil and Tullow Oil found petroleum reserves on the shores of Lake Albert.

North Kivu (French: *Nord-Kivu*) is a province bordering Lake Kivu in the eastern DRC; its capital city is Goma. To its north lies the province of Ituri, with Tshopo to the northwest, Maniema to the southwest, and South Kivu to the south. To the east, it borders Uganda and Rwanda. The province comprises three cities—Goma, Butembo, and Beni—and six territories—Beni, Lubero, Masisi, Rutshuru, Nyiragongo, and Walikale. North Kivu is home to Virunga National Park, a World Heritage Site that is home to the endangered mountain gorillas. Except for the heightened insecurity and isolation because of rebel activities, North Kivu shares similar demographics with Ituri. The province is politically unstable and has been one of the flashpoints of military conflicts in the region since 1998.

The 2018 or tenth Kivu Ebola outbreak began on August 1, 2018, when it was confirmed that four people had tested positive for the Ebola virus in the eastern region of Kivu^6–8^. The Kivu outbreak expanded to Ituri Province, after the first case was confirmed there on August 13^9^. This outbreak started just days after the end of the 2018 Ebola virus outbreak in Équateur province^10, 11^.

The affected province and general area was experiencing a military conflict, which hindered treatment and prevention efforts. The WHO’s Deputy Director-General for Emergency Preparedness and Response described the combination of military conflict and civilian distress as a “perfect storm” that could lead to a rapid worsening of the outbreak^12^. Owing to the deteriorating situation in North Kivu and surrounding areas, on September 27, the WHO raised the risk assessment at the national and regional level from “high” to “very high”^12^.

### Study population and sampling

The study population comprised adults aged ≥ 18 years living in the community as well as response team members. A 2010 estimate put the population of North Kivu at 5,767,945. With an annual growth rate of 3.2%, North Kivu’s population in 2019 was estimated at 7,658,406 for the general population and 5,360,884 for the ≥18 years group. With regard to Ituri, a 2005 estimate put the population at 4,037,561. Seventy percent of the population was aged ≥ 18 years (2,968,865). For 2019, the population was estimated as 6,275,305 for the general population and 4,392,714 for the ≥ 18 years group.

The response team consisted of more than 10,000 persons in different response pillars, namely surveillance, risk communication, social anthropology, and vaccination. Others included infection prevention and control, treatment and care, safe and dignified burial, as well as security, logistics, and administration.

A set of questions covering different thematic areas was developed to guide the focus group discussions with community members (male and female groups). The questions covered healthcare services in the community, awareness of and practices related to EVD, and assessment of the different pillars of the response interventions.

Trained research assistants with interviewing skills conducted the IDIs with community leaders, traditional healers, and Ebola survivors in each community. The IDIs were held with 17 purposely selected community/opinion leaders and 20 traditional healers. The IDIs were used to explore people’s opinions, views, and attitudes as well as practices and insights into the outbreak and their involvement in the response and other sociocultural factors that may have influenced their attitudes toward the response.

All interviews and discussions were tape-recorded, and detailed notes were taken simultaneously, including verbal citations. Tape-recorded interviews were transcribed according to standard rules. Observations were also recorded on paper and, together with the discussion and interviews, were triangulated with the quantitative data to arrive at conclusions.

### Data management

After review and correction, all interview transcripts were typed with a standard word processing package and converted into American Standard Code for Information Interchange (ASCII) text files. These were coded and sorted using the Atlas.ti program. Analysis of the qualitative data placed emphasis on the interpretation, description, and recording/writing of what was actually said. The transcription was first performed in the local language and then translated into English. While reviewing the transcriptions, phrases with contextual or special connotations were noted and extracted as illustrative quotes to complement the statistical data. To do this, relevant themes were developed for the coding and sorting of the qualitative data; Atlas.ti version 5.0 software was used to manage the qualitative data.

### Ethical considerations

The principle of “do no harm” was adhered to. Approval for the study was obtained at the provincial, local administration, community, and household levels while verbal informed consent was obtained from all individuals involved in the study. The WHO AFRO Ethics Review Committee provided ethical approval (AFR/ERC/2018/09.3). All researchers attended mandatory training, which included substantial discussion of the ethical issues in research. Fifty percent of the research assistants were females, ensuring same-sex interviews and moderation of focus group discussion sessions. The assistants were also trained and mandated to comply with gender sensitivity in the process of data collection and visits.

## Results

### Learning the profession

Traditional therapists learn the profession in different ways. Some learn from their parents. In many cases, the practice of traditional medicine has been in the family for generations. A traditional therapist from Glogu, a village in the Rwapara health zone, learned the skills from his grandmother. He said:

My grandmother used to send me to the scrublands to look for plants. She would explain everything to me and tell me the use of each plant. After she passed away, I took her place. I have been practicing since 2013.

Another healer from the same village, who also learned the skills from a relative, has been practicing for over 50 years. In Beni, we met with a traditional therapist born into a family of healers. He was taught by his grandparents in Lubero before moving to Beni. Two other traditional therapists from Beni were trained in an association that promotes the Nandé culture. This association covers other localities in the Beni area. We encountered two other traditional therapists who approached the profession within the framework of an association, the Association de Développement Culturel Yenandé, where they were taught natural medicine.

Some traditional therapists graduated from higher education institutions before learning their current profession. They were trained at a center. This was the case of a person from Butembo who first graduated from the Higher Institute of Applied Chemistry and then studied at the Institut National de la Santé et de la Recherche Médicale, the French National Institute of Health and Medical Research. Another traditional therapist from Butembo told us how he learned the profession:

If I am a healer today, it is because I inherited it from my parents. My father was a pastor; he practiced natural medicine, traditional in Rwanda, but he was also a trader, so he was often abroad. I remained a nurse in our center, here at home. After I finished my studies, my father wanted me to go to Rwanda to learn natural medicine from the Adventists. But home is where I learned what I know as a traditional therapist. Thus, I can say I inherited becoming a healer.

In Butembo, we met a traditional therapist who works within the framework of MENAMO, an association aimed at modernizing natural medicine, although his father had taught him the profession.

### Diseases treated

The traditional healers we interviewed said that they treat several illnesses: *Karo, Karufo* (poison, *sumu*), hemorrhoids, sexual dysfunction, sexual impotence, typhoid, liver disease, risk of miscarriage, gynecological issues, witchcraft attacks, childhood diseases, diabetes, paralysis, fractures, panic attacks, sterility, and appendicitis, and some also treated livestock. A traditional therapist from Gblogu, in the Rwanmpara Health Center in Ituri specialized in treating cows, witchcraft attacks, and children’s diseases—bloating, malnutrition, and so on. He claimed to also treat diabetes, paralysis, fractures, panic attacks, sterility, and appendicitis.

### Involvement in the response to the EVD outbreak

The coordination of the response to the EVD epidemic has involved traditional therapists in both North Kivu and Ituri. Traditional therapists have thus been trained and vaccinated. The training covered several aspects of the response. For example, a traditional therapist from Butembo was educated in infection prevention and control (IPC) and active case surveillance. He told us of the importance of his IPC training:

The IPC training has helped us a lot to protect ourselves. We wear personal protective equipment like gloves and aprons. We palpate the patients. They often vomit. Sometimes, we massage them, so we touch the patients. We wear gloves. But there often is a deficiency of gloves here.

It is in this context that many of them have collaborated with the health and response systems. For example, a traditional therapist from Bembo spoke about his role in the response:

I play an important role in the fight against Ebola. It is why I am still working here. I am still doing sensitization so that people understand that Ebola exists. Moreover, I send patients to a health center when they come to me presenting signs that, as we were instructed, may indicate a suspected case.

The collaboration with health personnel was usually beneficial, according to the same traditional therapist:

We collaborate with the health workers. Indeed, every day we report to them on the patients we see, especially the new patients. We give information on the disease as it has been taught to us and we describe the patients’ symptoms.

According to him, he referred any patients he was suspicious of to a health center:

For example, when the patients came, or even if they come now, if they show more than three signs, we send them back to the hospital for testing to check if they are not suffering from other diseases.

### Changes in professional practices

Traditional therapists involved in the EVD outbreak response have changed their professional practices. They have taken measures to protect themselves against the disease and to prevent infection. They have set up a sorting system where they measure the temperature of every person who comes to see them. A traditional therapist talked about the measures he takes in his daily practice:

I can say the changes begin with the reception. We have, for example, the sorting area. When the patients arrive, first we take their temperature, then they wash their hands, and finally we disinfect their shoes. During consultation, we keep ourselves at a distance to avoid contact or saliva if the patient coughs, for example. We wear gloves, and so on.

Collaboration with the health system occurs in different ways: raising awareness among patients, clients, and the general population; active surveillance of cases; referral of suspected cases to health institutions; and the adoption of infection prevention measures. Collaboration with the field response coordination has had an impact on the activity of traditional therapists in terms of patient attendance. Owing to the fear of EVD, patients’ presence in health services has decreased. Thus, traditional therapists who have been involved in the response have seen a drop in attendance at their workplaces. A traditional therapist in Butembo said:

The epidemic has really contributed to the drop in our clientele. We don’t receive as many patients as before. They are afraid of the epidemic. Besides, they believe that when you are already affiliated with the response team, you are also a member of the response. They think that the response teams are transmitting the disease.

Another traditional therapist in a village not far from Butembo made the same observation:

Before, we used to receive many clients here. Now, we receive only five clients per week. Before the epidemic it used to be around 20 clients a week. The patients are getting ideas. They stopped coming since we started to cooperate with the people responding to the epidemic. The patients ran away from us. There are only a few who stayed. There are patients that we will refer to the health center. This is not appreciated by the clients since the surge of the epidemic here. The last case was on September 2, 2019. We wanted him to go to the ETC. He went to another facility. He thought he had been poisoned. We followed up. We took action to make him go to the ETC. We got him to the ETC. But it was too late. He died there.

The workplace of this traditional therapist was equipped with a sorting system. In addition, he had a management algorithm and educational posters on EVD. However, not all traditional therapists were involved in the response. Not all of them had been trained and vaccinated, although all the healers we met had heard of EVD, some since the outbreak of the epidemic and others long before it happened. A traditional therapist from Gblogu said:

I heard about Ebola on the radio. When I go to the hospital, I see handwashing facilities. I don’t know if it’s for Ebola.

A traditional therapist from Butembo said that he looked for handwashing amenities and personal protective equipment for himself for 11 months. He then received personal protective equipment and other infection prevention equipment from EVD response officials. However, some of his clients stopped visiting him when they saw the handwashing amenities and the sorting system at his office. He used to see 10 to 15 patients a day. At the time of the interview, he was seeing only two patients a day, at most. He said that his center was affected because he had to pay the rent and staff salaries. He no longer had the resources to maintain it. He added:

There are centers that have closed their doors, both health centers and centers run by traditional therapists. We have not closed. It is serious to do so. But some centers have closed for fear of the disease without any constraints.

He also told us about the free healthcare policy, which has affected traditional therapists as some of their potential clients have left to go elsewhere. A traditional therapist from Butembo said he had asked to be trained. He had a 10-day IPC training course in two five-day sessions. The training sessions were spaced, and, for him, the second session was more significant. He was able to get a one-day training session for the rest of the staff at his treatment center. The training focused on EVD and how to prevent it. He spoke about his involvement in the response to the EVD epidemic:

I am involved at home in my center because I do awareness-raising with my patients. I talk to them about the risks they run if they don’t take the preventive measures against the disease. I was involved in active case finding from here. The people from the response team came to ask me to be active in case finding. I have been collaborating with the response team ever since. So, we are vaccinated here. We went to a vaccination site on our own.

Some traditional therapists participated in the response on their own initiative.

### Personal initiative for involvement

A few traditional therapists personally decided to join the response. Overall, the traditional healers we met in Butembo, Beni, and Bunia were asked to participate by the response coordinators of their villages or towns. However, one of the traditional therapists in Beni decided to contribute to the response on his own volition, based on the information he had about EVD. He is the only one we interviewed whose center was not impacted by the EVD epidemic. He is a traditional therapist but also a fetishist; therefore, he is feared. Clients come to him when they are not satisfied with the health centers. He had heard about EVD and the epidemic but had to search for information on the disease. He said:

Personally, I thought it was good to be close to people who are informed about the disease. I went to see people from the response team who were doing sensitization in the neighborhood. There were health workers among them. I asked questions. I learned that Ebola is a dangerous disease. As a traditional therapist, I have many patients. I had to find information about the disease. I searched in every way I could. I downloaded documents and consulted information on social networks about the epidemic. So, in my daily work, I took precautions.

He took precautions based on the information he had access to and diagnosed without touching the patient. He also used divination in the diagnosis, which allowed him to know if the person had a disease or if they were a victim of a sorcery attack. Based on the information he gathered on EVD, he set up a handwashing amenity in his workplace and referred five patients (three men and two women) to the ETC in Beni. The cases were confirmed and treated. All of them were cured, according to him. He visited his patients to encourage them. He told us what he did in each case:

There were cases that frightened me. I contacted the people in the fight against Ebola each time. I always followed up. They were taken to the ETC. They tested positive for EVD. I was following these patients since they had been taken to the ETC because of me. I used to visit them. It was reassuring for them to see me. All of them were treated and cured. Afterward, the three men were hired at the ETC. When they get their salary, they call me. Then, they offer me part of their salary and thank me. As for the two girls, they went home when they were discharged.

A traditional therapist lost relatives to EVD in Beni and decided to become involved in the response. She is also a radio host. She does research and broadcasts information on EVD. As a traditional therapist, she transferred three people to the ETC, one of whom was cured. She procures personal protective equipment herself, such as gloves and sanitizer gel. She has also set up a sorting system at the entrance to her office. She says she has not received any training by the response team. Although she is not involved in the response activities, she produced an awareness-raising video on her own, based on three key issues: the need to consider the birthplace of those who visit families, the recommended behavior when facing a person with major symptoms of EVD, and the importance of a rapid recourse to the ETC. The video is based on a case that addresses two of these aspects. She said she sent a copy of the video to some of the leaders of the response. She expressed a desire to work with the EVD response. Although she has not been formally trained, she knows that many traditional therapists have been. Another traditional therapist from the same association also expressed his willingness to participate in the response. Neither of them has been vaccinated. They expressed resentment for not having been involved in the response activities by the authorities.

### Involvement not appreciated

For some traditional healers, participation in the response activities was undesirable. The members of an association of traditional healers did not appreciate the presence of the multinational response team in Beni. One of them said:

The collaboration with the traditional healers has been abandoned. We were not consulted in the response to the Ebola epidemic. Our ideas and opinions are not considered by those responsible for the response. We have even been asked to stop providing care. We have abandoned our structures and become unemployed. We have been left without work.

Nearly 70% of the members of his association have benefited from training, but he believes that they were abandoned after the training. Usually, it is the level of involvement that is not appreciated. Thus, the members of a traditional therapists’ association in Beni claimed that they would like to be in all the response teams. Some of them felt that their participation was limited, as if it were unwanted by those in charge of the response. One of them emphasized the importance of the presence of traditional therapists at the ETC, even if they do not carry out activities there:

The presence of traditional therapists can reassure patients. If the patient does not find someone to trust at the ETC, he may worry. The Ministry of Public Health has not considered traditional therapists as healthcare providers.

Another traditional therapist said:

We were forced to work in secret. The patients also preferred to come in secret. When a traditional therapist refers a patient to the ETC, people start to fear him. It is as if the traditional therapist who transfers a patient to a health center abandons him. The traditional therapist is the patient’s last resort.

Yet another deplored the closure of traditional therapists’ offices:

Many of our offices have been closed. We were asked not to treat people. We were killed in this way. We were killed by unemployment. Ebola has exhausted us.

A traditional therapist in Butembo said that, at the beginning of the epidemic, traditional therapists were seen as enemies of the response. Yet he noted that traditional therapists have the trust of the people and that excluding them from the fight against EVD is not positive: *“We have the communities’ trust, and our orders are followed by the people. They listen to us.”*

According to him, some community members left town out of fear as a formal exclusion had no effect on the community’s choices. He thus contributed to the fight against the EVD epidemic by referring patients to health centers:

When we have suspected cases, we alert. There was a case of a 25-year-old woman. I was afraid and I informed the people who deal with Ebola here. They came and after some discussion, they took the patient away.

While the traditional healers we interviewed were involved in the response to the EVD outbreak, none of them claimed to have found a drug to treat the disease. However, some of the traditional healers wanted to use the outbreak as an opportunity for self-promotion. They sought to get involved in the fight against the epidemic by remaining outside the response system put in place by the Congolese state and its partners.

### Involvement for self-promotion

While some radio workers participated in the response, others sought to use the epidemic as an opportunity for self-promotion. In Beni, a group of traditional therapists spoke on the radio to say that they had found an effective drug for the treatment of EVD. This drug was named Eboline for marketing reasons, as it contains the name of the disease. We obtained this information from various interviews. However, we did not meet any members of this group during this study. The national authorities intervened to stop the dissemination of this information. This is how a member of a youth association in Beni described it:

Traditional therapists have said that with herbal medicine, they can treat EVD. It is a liquid product. But I don’t know the composition. Most of these herbalists have Eboline, which they give to the sick. The information is passed on by word of mouth in a clandestine way. Some traditional therapists have tried to talk on the radio about the product. But the authorities intervened to stop the broadcast. This is a radio station from Beni. It is a local radio station.

### Traditional therapists’ perspectives on their participation

A traditional therapist said:

The Ministry of Health must keep an eye on traditional healers. Because within this response, traditional therapists are neglected, disregarded; they are forgotten. Apart from a few who are specifically involved, not to say clandestinely, traditional practitioners are generally not involved.

The traditional therapists considered their involvement indispensable. A traditional healer in a village not far from Butembo requested a lot of information about the ETC from the authorities. He said that posters were not enough as not everyone understood them. He said:

People need to know that we don’t kill at the ETC. You must take people to a treatment center and an ETC. Not everyone can understand that at the same time. There was a caravan here organized by the people of the response. It was very useful. Everyone is talking about it. This way people can see and understand. There are healed people here. We need to look for those who have recovered and involve them in the awareness campaigns, mainly those who communicate well. We need to show people that Ebola can be cured.

A traditional therapist from Beni believes that people involved in the fight against EVD are earning too much money compared with what they used to earn. According to him, people should be hired on a voluntary basis. He said all traditional healers should be involved—especially the ones considered fetishists, as they were in touch with many people.

Some traditional therapists mentioned a lack of transport as a handicap to participation in the response, particularly in awareness-raising activities. Proposals to improve the response to the EVD epidemic included:

- Dealing with other diseases, such as tuberculosis and leprosy, not just EVD
- Selecting the right response agents
- Carefully selecting the NGOs involved in the response and demanding concrete actions from them
- Involving traditional therapists in the response
- Taking traditional medicine into account, as many patients resort to traditional therapists
- Improving the way awareness is raised
- Involving the state in awareness-raising to prevent epidemics in the country
- Having a model for community outreach to ensure better engagement
- Involving the young, religious leaders, neighborhood chiefs, association chiefs, heads of households, heads of living church communities (LCCs), members of LCCs, school principals, class leaders, pupils, and students
- Conducting information caravans on EVD
- Displaying awareness materials in public places

## Discussion

Traditional healers play an important role in the communities of North Kivu and Ituri. They are among the population’s first choices in the case of an illness. They work individually or in associations and, in both cases, have treatment centers. Some associations act simultaneously in several health zones. In general, traditional therapists learn from their relatives, usually their parents or grandparents, and view their profession as an inheritance. However, some are taught in traditional therapists’ associations and there are others who graduated from higher education institutions before learning their current profession.

Some of the traditional healers said they could treat several illnesses, from hemorrhoids to panic attacks. There are diseases for which traditional medicine is used exclusively. Traditional healers have been involved in the response to the EVD epidemic in several ways. In some cases, their involvement was initiated and organized by the response teams. Thus, some traditional healers received specific training on EVD, its prevention, and how to behave when faced with a suspected case, that is, referral to an ETC. After this training, the traditional therapists introduced changes in their daily professional practices. They adopted the use of gloves, installed handwashing amenities, and began sorting patients in their workplaces.

The treatment of certain diseases is under the exclusive realm of traditional medicine, which is seen as being able to heal several illnesses^13^. Most of the traditional healers claimed that patients came to them after going to health centers. A healer from Beni said that patients went to traditional therapists first when they did not believe they could be treated by modern medicine. Patients usually came from the traditional therapists’ local communities, but also from other regions. A healer from Butembo said he had patients from Goma, from other African countries like Rwanda, Uganda, and Kenya, and even some from countries outside the African continent, like India.

Traditional healers treat a wide range of diseases. In the Central African Republic, a healer said he could treat 150 diseases of various kinds with plants^14^. In Cameroon, after self-medication, recourse is made to traditional medicine or therapists as the first and second resort. In Côte d’Ivoire, people resort to traditional medicine^15, 16^, and the same is true in Gabon^15, 17^ and Cameroon. Traditional medicine is also present in the WHO’s programs. From 2000 to 2010, the WHO celebrated a decade of traditional medicine, a practice widespread in all African countries. Traditional medicine is also practiced in many countries around the world^18^.

In the EVD outbreak in the DRC, participation in the response was based on traditional therapists’ own initiative. Some sought information about the disease on their own to protect themselves but also to contribute to the response. Some referred suspected patients to the ETC based on what they learned about the disease. Among those who became involved of their own accord, some raised awareness at local radio stations or through meetings within their community. A traditional therapist in Beni even made a documentary on EVD prevention with her own resources. One of the key messages of this production was the importance of a rapid recourse to the ETC.

In certain cases, however, this involvement was not appreciated by the response authorities, who took measures against the traditional therapists, including the closure of their workplaces. The traditional therapists deplored the attitude of the response authorities who demanded the closure of their offices and, as a protest, many transformed their homes into workplaces. However, some traditional therapists saw the outbreak of EVD as an opportunity for self-promotion. They spread information about the discovery of a curative drug named Eboline. Measures were taken to prevent them from broadcasting this information through community radio stations. This was also observed to be the case during the EVD outbreak in West Africa.

In Mali, traditional healers were involved in the response to the EVD epidemic^19^. However, the importance of traditional and religious practices has often contributed to the spread of EVD in West Africa in a context marked by a lack of information about the disease^20^. In some cases, traditional therapists have not facilitated the response to the EVD epidemic^21^. Some have claimed to be able to treat EVD; this is discussed below. Therefore, sensitization activities aimed at traditional therapists have been implemented by the EVD response programs to qualify healers to distinguish potential EVD patients from other patients. Without this training, traditional therapists can become a source of misinformation to the population, especially in remote areas^20^.

In some countries, traditional healers have no links with the health system in the fight against EVD^1^. Besides, little research has focused on the involvement of traditional healers in the response to EVD. The traditional health professionals who have not been involved want to be involved and want specific training. Half of those who were cured of EVD in Sierra Leone resorted to traditional therapists. However, they also resorted to a variety of treatments, including self-medication with modern medicines and consultation with a modern medical professional. Besides those who were cured, women, especially pregnant and lactating women, resorted to traditional medicine^1^. In Africa, more than half of the population uses traditional and complementary medicine^2^. In West Africa, 70%–80% of the population depends on traditional medicine^3^.

During an EVD outbreak in Uganda, community members asked a traditional healer to identify the origin of the disease, but the experiment was not successful. However, some cultural beliefs and practices were likely to contribute to the response^22^. In Beni, an association of traditional therapists was involved in community dialogue on the vaccine because of the increase in alerts, community-based surveillance, and IPC activities for three months.

At the beginning of the epidemic in the DRC, traditional healers were seen as enemies of the response as their involvement in the fight against EVD was seen as a source of problems^21^. Many of them left because of this adversity. In some countries, the involvement of traditional healers in the response has been prohibited. For example, in Sierra Leone, they were considered to be stimulating the epidemic. Nevertheless, organizations have engaged in dialogue with traditional therapists to involve them in the response without marginalizing or blaming them. Hence, the emphasis has been on the need to further involve traditional healers and midwives as part of the frontline staff in the response at the local level^23^.

Traditional healers generally learn their skills from relatives, usually their parents or grandparents. Some are educated in traditional therapists’ associations. Others graduate from higher education institutions before learning their profession skills. They claim to treat several illnesses. Inhabitants of the provinces of North Kivu and Ituri rely on traditional therapists to treat illnesses that are considered exclusive to the realm of traditional medicine.

Some traditional healers work individually, and others work in associations. Traditional therapists have been involved in the response to the EVD epidemic in several ways. Generally, their involvement was initiated and organized by the response teams. The response teams educated them on EVD, prevention measures, and what to do when dealing with a suspected case—referring the patient to an ETC. This training led to changes in the therapists’ practices and workplaces as they adopted the use of gloves, installed handwashing amenities, and implemented procedures to categorize their patients.

For other traditional therapists, involvement was the result of their own initiative to find information about EVD. Some referred suspected patients to an ETC. Of their own accord, some raised awareness on community radio stations and organized sensitization meetings in their communities. A traditional therapist from Beni produced a documentary on EVD, which discussed its prevention and the importance of going to an ETC. However, the involvement of traditional therapists was not always appreciated by the response authorities, who acted against them by closing their workplaces. National authorities demanded the closure of traditional therapists’ workplaces, leading most of them to convert their homes into offices.

Some healers saw the outbreak of EVD as an opportunity for self-promotion. They shared rumors of a cure they had discovered, a drug named Eboline. Measures were taken to prevent them from spreading this information. These traditional therapists were particularly active in Beni. Traditional therapists proposed to improve the response vis-à-vis their involvement and the inclusion of traditional medicine. Some proposed to address other diseases, such as tuberculosis and leprosy. Others asked for the involvement of young people, religious people, neighborhood chiefs, heads of mutual societies, heads of LCCs, members of LCCs, school principals, heads of classes, pupils, and students, and for information on EVD.

## Data Availability

The data that support the findings of this study are not publicly available as they contain information that could compromise the privacy of the research participants. The data are available from the corresponding author (Joseph Okeibunor) upon reasonable request.
